# The Effects of Social Perception on Moral Judgment

**DOI:** 10.3389/fpsyg.2020.557216

**Published:** 2021-03-23

**Authors:** Wen Ying Jin, Ming Peng

**Affiliations:** ^1^School of Psychology, Central China Normal University, Wuhan, China; ^2^Key Laboratory of Adolescent Cyberpsychology and Behavior, Ministry of Education, Central China Normal University, Wuhan, China

**Keywords:** moral judgment, social perception, deontology, utilitarianism, warmth, competence

## Abstract

When people express a moral judgment, others make inferences about their personality, such as whether they are warm or competent. People may use this interpersonal process to present themselves in a way that is socially acceptable in the current circumstances. Across four studies, we investigated this hypothesis in Chinese culture and showed that college student participants tended to associate others’ deontological moral judgments with warmth and utilitarian moral judgments with competence (Study 1, *M*_age_ = 21.1, SD = 2.45; Study 2, *M*_age_ = 20.53, SD = 1.87). In addition, participants made more deontological judgments after preparing to be interviewed for a job requiring them to be in a warm social role, and more utilitarian judgments after preparing for a job requiring them to be in a competent social role (Study 3, *M*_age_ = 19.5, SD = 1.63). This effect held true in moral dilemmas involving different degrees of hypothetical personal involvement, and appeared to be mediated by the perception of others’ expectations (Study 4, *M*_age_ = 19.92, SD = 1.97). The results suggest an important role for social cognition as an influence on moral judgments in Chinese culture.

## Introduction

Moral judgment is the evaluation of a certain behavior as good or bad, or as right or wrong. The goal of moral psychology is to clarify why individuals make the judgments they do about moral issues. Research on moral judgments has been especially influenced by the two most important normative ethics theories of the last several centuries, that is deontology and utilitarianism. Both theories prescribe logic for determining the morality of behavior. A deontological perspective is one that evaluates a behavior as right or wrong based on the action itself. A utilitarian perspective is one that evaluates a behavior as right or wrong based on the outcome of the action. In the field of moral psychology, the “moral dilemma” is a classic moral judgment problem that has been used in numerous studies to discover people’s tendency to make moral judgments in various situations.

### Theory and Models

Existing models of how people make moral judgments are organized around two basic objectives (Guglielmo, 2015). Information Models have the goal of identifying the specific information content that forms the basis of people’s moral judgments: the various aspects of the behavior, the extent to which the actor’s relevant characteristics lead people to believe that the actor is responsible and reprehensible, such as the causation of agents’ behavior (Lagnado and Channon, 2008), the actor’s degree of intent (Darley and Shultz, 1990; Ohtsubo, 2007; Gray et al., 2012), and the actor’s reasons, motivations, and beliefs (Suls and Kalle, 1978; Nelson-Le Gall, 1985; Zelazo et al., 1996; Young and Saxe, 2009; Tannenbaum et al., 2011; Inbar et al., 2012). By contrast, Processing Models have the goal of determining the psychological processes that produce moral judgments, including the extent to which these judgments are driven by intuitive, emotional processing or by thoughtful, rational processing.

These theories on moral judgment focus on the “antecessors” of moral judgment (i.e., the information taken into account and how the information is processed), but they pay little attention to understanding the “consequences” of moral judgment, especially the social consequences (Rom et al., 2017).

### Social Perception Based on Other’s Moral Judgments

What are the consequences when people make their moral judgments? One important consequence may be that people’s judgments of moral dilemmas affect how others view them. Haidt (2001) argued that moral judgments are essentially social in nature because they convey important information about the person making the judgment. The assumption is that observers make inferences about a person’s character based on the person’s moral judgments.

Numerous studies have found that people are sensitive to the psychological factors that drive others to make moral decisions (Weiner, 1985; Cushman and Mele, 2008; Pizarro and Tannenbaum, 2011). Recent works have also shown that bystanders make inferences about others’ personalities based on the moral judgments they make (Uhlmann et al., 2013; Kreps and Monin, 2014; Everett et al., 2016; Sacco et al., 2017). According to research, people who make deontological decisions in moral dilemmas are rated as more empathetic and as having higher moral qualities than those who make utilitarian decisions (Uhlmann et al., 2013), and those who express utilitarian views are considered less moral than those who express deontological views, sometimes even less moral than those who express no clear views at all (Kreps and Monin, 2014). People who make deontological judgments are also more likely to be chosen as social partners and are considered more moral, likeable, and trustworthy (Everett et al., 2016; Sacco et al., 2017), including being more trustworthy in economic games than those who make utilitarian judgments (Everett et al., 2016). In several experiments conducted by Rom et al. (2017), participants made inferences about how emotion and cognition affected the moral decision maker’s judgments, and they used this information to infer whether the decision maker was warm or competent. Specifically, participants rated people who made deontological judgments as relatively warm and enthusiastic, whereas people who made utilitarian judgments were rated as relatively competent.

Some researchers (Uhlmann et al., 2015) have proposed human-centered explanations for moral judgments, which focus on individuals rather than behaviors as the unit of moral evaluation analysis. With regard to moral judgment, people are more like intuitive virtue theorists rather than deontologists or utilitarians, who each describe people in a one-sided way. There is growing evidence that individuals are fundamentally motivated to evaluate others on a moral level—people quickly and easily attribute good or bad moral traits to others at an early stage of interaction, with limited information (Goodwin et al., 2014).

### Social Situation Influence Moral Judgments

Rom et al. (2017) also raised questions about whether people who are making moral judgments are aware that others may judge them accordingly, and, if so, whether people strategically shift their solution of the dilemma to create an ideal impression. According to the theoretical framework of social cognition (Bandura, 2001), it is usually the interaction of situational factors and individual characteristics that leads people to engage in certain behaviors and to make certain decisions. Moral judgments should also be influenced by social cognitive factors, but most previous studies (Guglielmo, 2015) have ignored this issue. At the very least, there appears to be a conformity effect in moral judgment making.

That is to say, participants appeared to change their publicly presented moral judgment, suggesting that there can be proactive processing in moral judgment (Uhlmann et al., 2009; Liu and Ditto, 2013), and the judgment of moral issues is sensitive to social impact. Similarly, Kundu and Cummins (2013) asked participants to make moral judgments about a range of dilemmas, either alone or in a group that included three confederates. The results showed a significant conformity effect: compared to participants who made moral judgments by themselves, those in a group made more judgments that were consistent with the other group members’ judgments, even though those judgments were contrary to common sense.

Thus, we speculate that when people make moral judgments in real life, they engage in social cognition and will consider and integrate other information from the outside world as needed to adjust their judgments. In fact, if people believe that conformity with others can maximize the expected value of decision-making, conformity can be seen as a rational choice. In social interactions, people no longer think only about the event itself, but also plan and evaluate the consequences of their actions, which can be adaptive. It can be inferred that higher-order social cognitive processes are likely to transcend low-order affective and cognitive processing of dilemma judgments (Rom et al., 2017). Behavior in a social situation has both the significance of fact evaluation and the characteristics of value evaluation. In social behavior, task-oriented motivation (focus on task), and expressive motivation (display of self-related characteristics, impression management) exist simultaneously. Individuals are motivated to engage in active impression management when they realize that their behavior is being or may be evaluated by others. If individuals want to let others know who they are, they need to package information about themselves and present the information in a concise way to make the desired impression on others. According to Leary and Kowalski (1990), all behaviors can be regarded as self-presentation behaviors. Moral behavior is no exception.

Everyone inevitably plays different social roles at different times and places, each of which has corresponding social expectations (Callero, 1994). In the field of social cognition, numerous studies (Fiske et al., 2002; Abele et al., 2008; Cuddy et al., 2008) have confirmed the existence of people’s common stereotypes and prejudices toward various groups. As individuals and groups in social situations are evaluated and recognized, they are motivated to convey information to the outside world through their behaviors and decisions (such as decisions regarding moral dilemmas). Especially as a link in the formation of cultural norms, moral judgment not only has important adaptive significance for individuals and groups but also plays an important role in the production of moral ethics and cultural norms in the process of interaction. However, there is still a lack of research on the influence of social cognition and social perceptions on moral judgments. The current study addresses this gap in the literature.

## Overview of Studies

As far as we know, only one study had investigated the moral judgments in the presence of situational expectations (Rom and Conway, 2018). They found that American and German participants had accurate meta-insight regarding the inferences of others, which draw about their personality from their dilemma judgments, and participants strategically shifted public dilemma judgments to present themselves as warm or competent in order to present situational favorable impressions.

We are also interested in whether such effects exist in Chinese culture. The moral status of specific social behaviors can vary widely across cultures. Previous studies have also suggested that there may be cultural differences in dilemma judgments. For example, those in collectivist cultures are more likely to consider additional contextual information when forming judgments, such as whether or not it is their place (or duty) to act (Gold et al., 2014). This relational consideration in turn leads to less reprimand of individuals who do not take action, and fewer character attributions of actions made in the absence of their broader contextual meaning (An and Trafimow, 2013). On the other hand, Chinese have more interdependent self-construals, one consequence of which is that they care more about the opinion of others (Markus and Kitayama, 1991), which makes it more likely that the Chinese will do what is expected of them.

Dimensions of basic social perception are culturally consistent. People’s daily lives require a quick and effective impression of most other individuals. In studying how people perceive and understand each other, there appear to be two dimensions that differentiate groups and individuals: warmth and competence (Rosenberg et al., 1968; Wiggins, 1979; Peeters and Czapinski, 1990; Peeters, 1992, 2008; Phalet and Poppe, 1997; Paulhus and John, 1998; Dubois and Beauvois, 2005; Judd et al., 2005; Abele and Wojciszke, 2007; Fiske et al., 2007; Cuddy et al., 2008). These studies have consistently shown that people hold significantly different stereotypes regarding the warmth and competence of target groups defined by occupation, ethnicity, nationality, religion, socioeconomic level, and gender. Surveys across different cultures found a stereotype content model with warmth × competence space mapping of social groups (Cuddy et al., 2007; Imhoff et al., 2013). We assume that there should be cross-cultural consistency in the social perception of moral judgments too.

When people perceive external expectations or requirements of a certain social role, they are likely to adjust their moral behavior accordingly to meet their needs or achieve goals. In the context of moral dilemmas, the person who makes the judgment and the person who evaluates the decision maker together create a form of social communication. We assumed that when Chinese participants perceive the target characteristics, they will strategically express moral judgments that conform to external expectations, so as to obtain expected results. The following four studies were carried out to test this assumption.

Study 1 examined participants’ inferences about the warmth or competence of an agent who made characteristic deontological or consequentialist judgments. Study 2 looked in the opposite direction to examine participants’ inferences about what kind of judgments would be made by an agent characterized as either warm or competent. In Study 3, we used several moral dilemmas to see if participants strategically shifted their moral judgments in response to perceived social factors (the social role they were striving to fulfill and the expectations of others). Study 4 repeated the steps of Study 3 and increased the measurement of participants’ prediction of external expectations to confirm the mediating effect.

## Study 1

Previous studies in Western countries have found that people evaluate those who make a particular kind of moral judgment as having varying degrees of competence or warmth. The primary concern of Study 1 was to see whether this same pattern of evaluation differentiation would be found in a sample of Chinese college students. We hypothesized that consistent with the results of earlier studies, participants will think that actors who make deontological moral judgments are more likely to be warm and that those who make utilitarian moral judgments are more likely to be competent.

### Method

#### Participants

This and studies to be reported next were carried out in accordance with the ethics principles of the Declaration of Helsinki. All participants signed an informed consent form for the experiment. Sample size in each study was determined before any data analysis.

Two hundred and thirty-two college students were recruited through WJX.cn and were paid¥2.5 for their time. Twenty-two participants’ questionnaires were eliminated from subsequent analyses due to too many blanks. Final *N* = 210 (118 females, 92 males). Mean age was 21.1 years (SD = 2.45).

#### Procedure and Materials

Participants were told that they needed to evaluate the different dimensions of a person’s personality in a questionnaire. The online questionnaire was arranged as follows: we firstly introduced an individual LM to participants (“LM is a college student of the same age as you, who has made some kind of judgment or choice in the face of several different situations, please make your evaluation of him/her according to his/her behavior”). Next, we presented three moral dilemmas and LM’s answers to them in a fixed random order. Then, we asked the participant to evaluate LM’s traits in each dilemma.

Three moral dilemmas were used in this study: Trolley, Lifeboat, and Crying baby dilemma.

Study 1 used the items adapted by Fiske et al. (2002) and employed by Rom et al. (2017) as the questionnaire evaluation topic, in which participants needed to rate LM’s warmth, competence, and morality. Participants expressed their evaluation on how well four warmth traits (warm, good-natured, tolerant, and sincere), five competence traits (competent, confident, independent, competitive, and intelligent), and one moral trait (moral) describe LM on seven-point scales anchored at 1 (not at all) and 7 (very much).

There were two kinds of questionnaires, one of which LM makes a biased consequentialist judgment on all dilemmas, and the other of which LM makes a biased deontological judgment. Participants would be randomly assigned to fill in one of the questionnaires.

### Results and Discussion

Firstly, questionnaire data were collated and descriptive statistics were conducted. This study concerned the differences in participants’ perception of the target object. Therefore, we averaged judgments into composites of warmth (α = 0.907), competence (α = 0.907), and morality (α = 0.722).

Ratings were submitted to a 2 (target tendency: deontology vs. consequentialism) × 2 (personality evaluation: warmth vs. competence) repeated measures ANOVA with the first factor between-subjects and the last factor within-subjects.

There was no main effect of personality evaluation, *F*(1, 208) = 0.027, *p* = 0.87, and η*_*p*_*^2^ = 0.0001, but main effect of target tendency was significant, *F*(1, 208) = 6.54, *p* = 0.011, and η*_*p*_*^2^ = 0.03. For targets who had shown a deontological tendency, the overall evaluation on warmth and competence they received (*M* = 4.44, SD = 0.70) is higher than those who hold consequentialist moral judgment tendency (*M* = 4.26, SD = 0.82).

**FIGURE 1:**
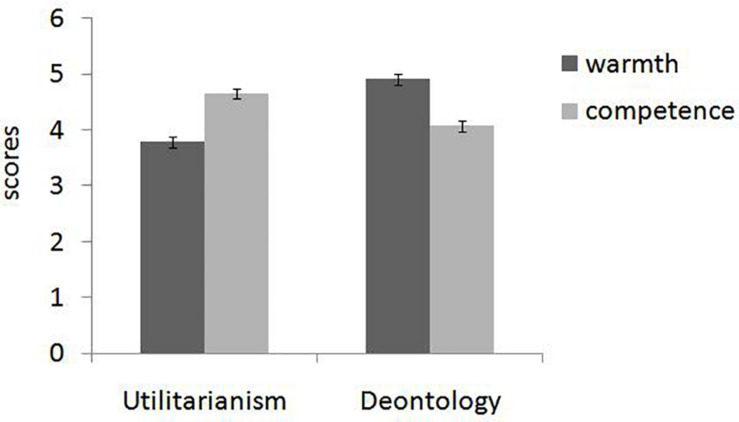
Agents who had shown a consequentialism tendency were considered more in accord with competence dimension rather than warmth dimension. Conversely, agents who had shown a deontological tendency were considered more in accord with warmth dimension (Study 1). Error bars reflect standard errors.

The two-way interaction between personality evaluation and target tendency was significant, *F*(1, 208) = 83.24, *p* < 0.001, and η*_*p*_*^2^ = 0.286. Simple effect analysis demonstrated that targets who had shown a consequentialism tendency were considered more in accord with competence dimension (*M* = 4.65, SD = 0.1) rather than warmth dimension (*M* = 3.79, SD = 0.1), *F*(1, 208) = 47.67, *p* < 0.001, and η*_*p*_*^2^ = 0.205. Conversely, targets who had shown a deontological tendency were considered more in accord with warmth dimension (*M* = 4.91, SD = 1.0) than with competence dimension (*M* = 4.07, SD = 1.0), *F*(1, 208) = 36.65, *p* < 0.001, and η*_*p*_*^2^ = 0.157 (see Figure 1).

In addition, the mean value of three grades on moral dimension items was calculated as a result of evaluation toward target’s moral score. Ratings were submitted to a target’s moral judgment tendency (consequentialism vs. deontology) *t* test. The results showed that the main effect was significant, *t*(208) = 6.77, *p* < 0.001, and Cohen’s *d* = 0.94; participants rated targets who had shown a deontology tendency (*M* = 4.78, SD = 1.30) as more moral than those who had shown consequentialism tendency (*M* = 3.57, SD = 1.30).

This study found that participants made more positive evaluations of individuals who held deontological moral judgments, both in the moral dimension and in the warmth and competence dimensions, which indicates that deontological moral judgments contain prosocial information to some extent. This result is consistent with the previous studies (Uhlmann et al., 2013; Kreps and Monin, 2014; Everett et al., 2016; Sacco et al., 2017).

The results of Study 1 also demonstrated that people believe that those who make deontological moral judgments are warmer, whereas those who make utilitarian moral judgments are more competent. This result is also consistent with previous research results (Rom et al., 2017). Based on Study 1, we know that Chinese college students, like participants in research conducted in the west, have a tendency to speculate about the personality characteristics that lead others to make certain moral judgments. However, this pattern has not been explored in the opposite direction to see if people assume that actors with different personalities make different types of moral judgments. This was further explored in Study 2.

## Study 2

The results of Study 1 showed that, as in other studies, Chinese college students made systematic inferences about the personality characteristics associated with deontological and utilitarian moral judgments.

In Study 2, we wanted to see if people with different personalities were seen by others as more or less likely to make different types of moral judgments. It was hypothesized that evaluators will think that a person who conforms to the warmth dimension will be inclined to make deontological moral judgments, whereas they will think that a person who conforms to the competence dimension will be inclined to make consequentialist moral judgments.

### Method

#### Participants

Forty-five college students (29 female, 16 males; *M*_age_ = 20.53, SD = 1.87) were invited into the laboratory to participate in Study 2 and were paid ¥5 for their time.

#### Procedure and Materials

Participants were first presented with several keywords about one of the basic dimensions of social perception. Then, they were asked to imagine a character X based on these keywords. Finally, they need to speculate on what judgments that character would make in several moral dilemmas.

All the words used in Study 2 were summarized by Cuddy et al. (2008). The moral dilemmas used are Trolley, Footbridge, and Lifeboat.

The final question of each dilemma was modified to ask participants to speculate to what extent X will think it is appropriate to make a consequentialist choice. They were going to rate the degree on the Likert seven-point scale, from 1 (totally inappropriate) to 7 (totally appropriate), with higher scores being closer to the consequentialist judgment and lower scores being closer to the deontological judgment.

### Results and Discussion

**FIGURE 2:**
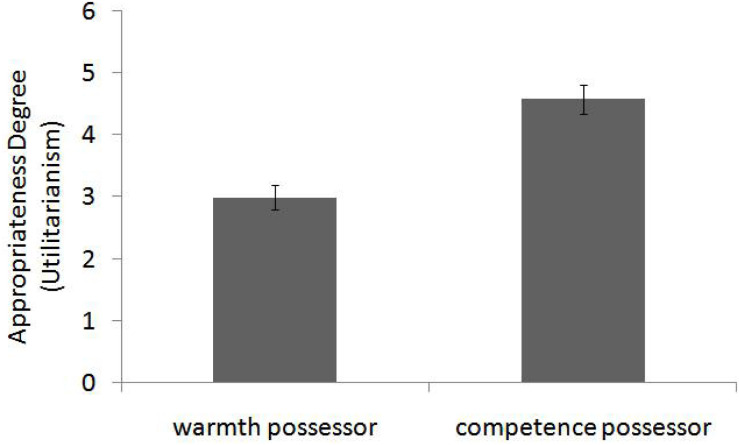
Compared with a warmth possessor, a competence trait possessor was regarded as more likely to think the consequentialism choices are more appropriate (Study 2). Error bars reflect standard errors.

Ratings were submitted to a personality (warmth vs. competence) independent *t* test and then found a significant main effect, *t*(43) = 5.15, *p* < 0.001, and Cohen’s *d* = 1.54. Compared with a person whose personality conforms to the warmth dimension (*M* = 2.99, SD = 0.95), a competence trait possessor (*M* = 4.58, SD = 1.12) was regarded as more likely to think the consequentialism choices are more appropriate (see Figure 2).

This result validates the previous hypothesis that participants do think that warm people will be inclined to make deontological moral judgments and that competent people will be inclined to make consequentialist judgments; this result further advances Study 1. On the basis of knowing that people’s character traits can be inferred from moral judgments, Study 2 shows that people’s tendency of moral judgments can also be inferred from their personality traits.

This raises the questions of whether people realize that others may judge them based on their moral judgments, and whether they will be able to strategically shift their judgments to create an ideal impression (Rom et al., 2017). Study 3 focused on these questions.

## Study 3

Having demonstrated that people infer personality traits based on others’ moral dilemma judgments in Study 1 and that they infer moral judgments based on personality traits in Study 2, in Study 3, we examined whether people will strategically shift their moral judgments based on information about others’ inferences. Specifically, Study 3 explored how people judge moral dilemmas when they perceive that they need to be in a certain social role with either warmth or competence as the core quality. It was hypothesized that the moral judgments will tend to be deontological when people realize that they need to strive to be in a warm social role. By contrast, their moral judgments will incline toward utilitarianism when they want to be in a competent role.

In Study 3, an interview situation was set up to test these hypotheses. Participants were required to prepare for a fictional job interview, in which they needed to work hard to get an offer. Each job they applied for corresponded to a social character whose core quality was warmth or competence. They were told that their performance in the job application process would determine their final payment. Hence, in order to ensure that the two characters in the experiment actually fit the warmth and competence dimensions, respectively, we conducted a pretest.

### Pretest

Forty-five Chinese university students were recruited (27 females, 18 males, *M*_age_ = 20.58, and SD = 1.85) to test whether participants prioritized warmth for receptionists of mental health center, and competence for restaurant managers.

We firstly introduced to all participants that warmth and competence are universal dimensions of social perception and each dimension was described by several labels. The words used here come from the summary of Cuddy et al. (2008). Then, they were asked to rate how important the traits of the two dimensions were to each character. Finally, participants rated the importance of the warmth and competence on scales from 1 (not at all important) to 7 (very important).

**FIGURE 3:**
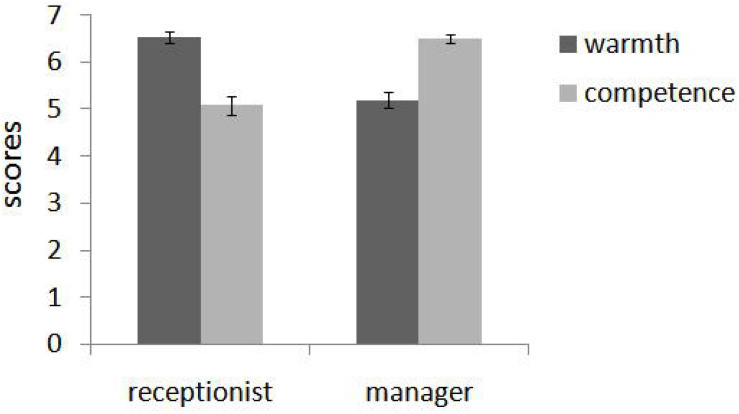
In pretest, participants rated warmth as more important than competence for a receptionist in a mental health center and competence as more important than warmth for a restaurant manager (Study 3). Error bars reflect standard errors.

These ratings were submitted to a 2 (role: receptionist of mental health center vs. restaurant manager) × 2 (trait: warmth vs. competence) repeated measures ANOVA with both two factors within-subjects. There was no main effect of either social role, *F*(1,44) = 0.131, *p* = 0.719, and η*_*p*_*^2^ = 0.003, or trait, *F*(1,44) = 0.317, *p* = 0.577, and η*_*p*_*^2^ = 0.007. However, we found a significant interaction, *F*(1,44) = 84.315, *p* < 0.001, and η*_*p*_*^2^ = 0.657. Simple effect tests demonstrated that participants rated warmth (*M* = 6.53, SD = 0.73) as more important than competence (*M* = 5.08, SD = 1.35) for a receptionist in a mental health center, *F*(1,44) = 59.77, *p* < 0.001, η*_*p*_*^2^ = 0.58, and competence (*M* = 6.51, SD = 0.59) as more important than warmth (*M* = 5.2, SD = 1.17) for a restaurant manager, *F*(1,44) = 45.00, *p* < 0.001, and η*_*p*_*^2^ = 0.51 (see Figure 3).

So, if people are going to apply to be a receptionist of a mental health center, they may make a more deontological judgment about the dilemma, given that the traits required for the role is warmth. They may make a more consequentialist judgment when they apply to be a restaurant manager, of whom the core personality is competence.

### Method

#### Participants

Seventy-one Chinese university students (30 females, 41 males; *M*_age_ = 19.5, and SD = 1.63) were invited into the laboratory to participate in this study.

#### Procedure and Materials

Participants were randomly assigned to one of two conditions (social role: receptionist of mental health center vs. restaurant manager). Firstly, they were told that they needed to go for an interview, and that their final payment would depend on how well they performed in the interview. The basic payment was ¥6, and the additional rewards ranged from ¥1 to ¥6. Then, they read a brief description of the job that they were applying for and took enough time to understand and prepare.

When the formal interview came, participants needed to first verbally describe their understanding of the key points of the job and their initial plans upon entry. After a short break, a questionnaire was filled out. The questionnaire included questions on moral judgment and simple intelligence test questions as fillers. After each moral dilemma, participants had a relatively long time to make decisions. They needed to rate to what extent they thought that it was appropriate to carry out a utilitarian behavior in each scenario. Participants used a scale ranging from 1 (not at all appropriate) to 7 (very appropriate), with higher scores being closer to a utilitarian judgment and lower scores being closer to a deontological judgment. The moral dilemmas used in Study 3 were Trolley, Footbridge, and Lifeboat dilemmas. We were also interested to see whether participants in the formal experiment, like those in the pretest, rated the importance of warmth and competence in the job differently. So, we increased measurement of this question at the end of the interview.

Instructions and questions were displayed on a computer screen at certain times, and participants responded with pen and paper. The reward they received was actually at random, not related to their answers.

### Results and Discussion

**FIGURE 4:**
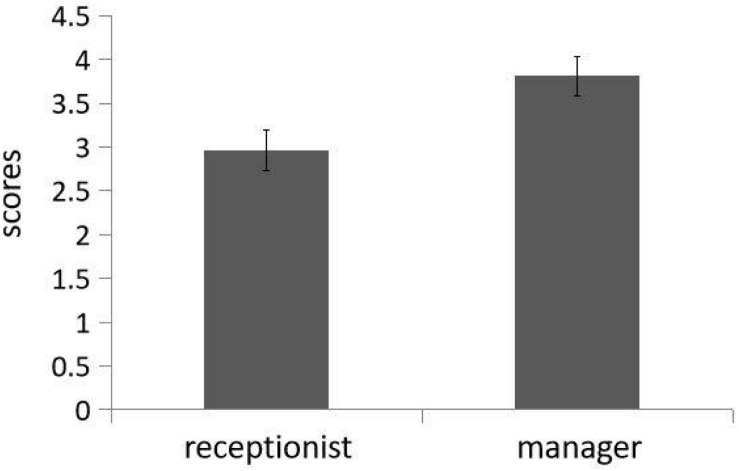
People who aspired to be restaurant managers exhibit more consequentialist tendencies than those who aspired to be mental health center receptionists (Study 3). Error bars reflect standard errors.

We submitted ratings to a social role (receptionist of mental health center vs. restaurant manager) independent *t* test and then found a significant main effect, *t*(69) = 2.67, *p* = 0.009, and Cohen’s *d* = 0.64. As predicted, people who aspired to be restaurant managers (*M* = 3.82, SD = 0.22) exhibit more consequentialist tendencies than those who aspired to be mental health center receptionists (*M* = 2.96, SD = 0.23; see Figure 4).

Moreover, Study 3 also re-examined participates’ ratings of the importance of warmth and competence in the two occupations.

**FIGURE 5:**
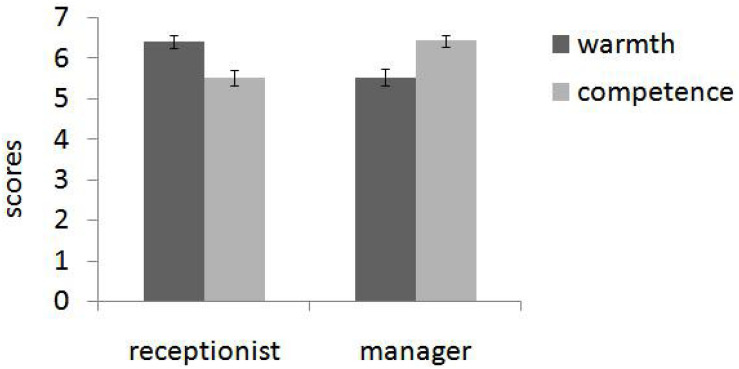
Participants in Study 3 rated warmth as more important than competence for a receptionist in a mental health center and competence as more important than warmth for a restaurant manager, which replicates the trend of participants’ evaluation in pretest (Study 3). Error bars reflect standard errors.

Ratings were submitted to a 2 (role: receptionist of mental health center vs. restaurant manager) × 2 (trait: warmth vs. competence) repeated measures ANOVA with the first factor between-subjects and the last factor within-subjects. There was no main effect of either social role, *F*(1,69) = 0.000003, *p* = 0.99, η*_*p*_*^2^ = 0.000003, or trait, *F*(1,69) = 0.013, *p* = 0.91, and η*_*p*_*^2^ = 0.0002. There was a significant interaction, *F*(1,69) = 31.41, *p* < 0.001, and η*_*p*_*^2^ = 0.313. Simple effect analysis demonstrated that participants rated warmth (*M* = 6.41, SD = 0.89) as more important than competence (*M* = 5.53, SD = 1.21) for a receptionist in a mental health center, *F*(1,69) = 14.46, *p* < 0.001, η*_*p*_*^2^ = 0.17, and competence (*M* = 6.43, SD = 0.83) as more important than warmth (*M* = 5.51, SD = 1.04) for a restaurant manager, *F*(1,69) = 17.07, *p* < 0.001, and η*_*p*_*^2^ = 0.19 (see Figure 5). This result replicates the trend of participants’ evaluation in pretest. Again, people do have different perceptions of warmth and competence in the mental health center receptionist and restaurant managers.

The results of Study 3 conformed to the hypotheses. When people perceive that others have expectations of them, and strive to be in a social role that conforms to the warmth dimension, their moral judgments are inclined to be deontological. When people strive to be in a social role that conforms to the competence dimension, their moral judgments are inclined to be consequentialist. This conclusion suggests that moral judgments, like many other social judgments, are sensitive to environmental factors and social influences, rather than reflecting a stable individual approach to moral decision-making.

## Study 4

The results of Study 3 suggested that people do make different moral judgments when faced with the demands of different social roles, consistent with the assumption that moral judgments are sensitive to environmental factors, but what was the reason for this effect? Those effects we found in Study 3 are driven by a number of motivations such as making a good impression to others, obtaining approval, and finally getting a job offer. In social interaction, people need to identify the ideal expectations of their evaluators, then perform in accordance with this expectation. Hence, we assumed that the perceived external expectation would be an important mediator of the effect of social role on moral judgments. Therefore, Study 4 had two objectives: to examine whether the effects in Study 3 could be replicated in a new sample and to test the mediating effect of perceived external evaluation.

### Method

#### Participants

Sixty-four Chinese university students (41 females, 23 males; *M*_age_ = 19.92, SD = 1.97) participated in Study 4.

#### Procedure and Materials

Procedure and materials of this study are similar to those of Study 3. Once again, a new group of participants were asked to accomplish the task of job applying. They had to make efforts toward adopting either a receptionist of mental health center (a warm role) or a restaurant manager (a competent role). We again measured participants’ moral judgments in the context of different social perception interventions. However, only one dilemma (Footbridge) was used here. In addition, based on the experimental process of Study 3, Study 4 added a cognitive question of participants on the recruiters’ ideas at the end. We asked: “and what do you think the recruiter is looking for in your response to that dilemma question?” Then, participants should rate it on a scale from 1 (not at all appropriate) to 7 (very appropriate), with higher scores being closer to the deontological judgment and lower scores being closer to the consequentialist judgment. Instructions and questions were displayed on a computer screen at certain times, and participants responded with pen and paper. The reward they received was actually at random, regardless of their answers.

### Results and Discussion

**FIGURE 6:**
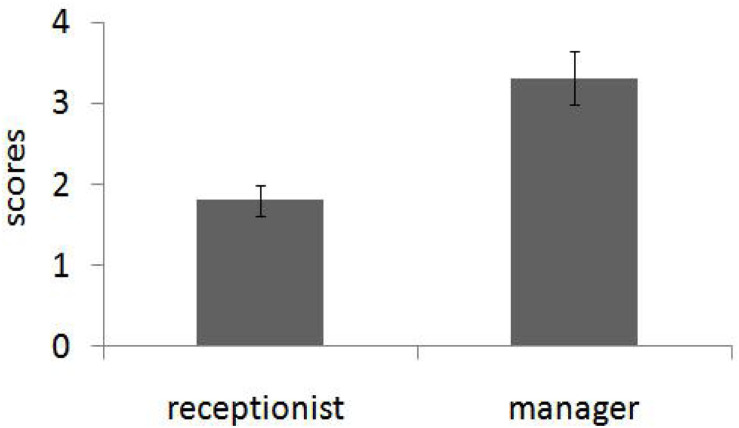
Participants who aspired to be restaurant managers (competent role) exhibit more consequentialist tendencies than those who aspired to be mental health center receptionists (warm role), which is consistent with the trend of results in Study 3 (Study 4). Error bars reflect standard errors.

We submitted ratings to a social role (receptionist of mental health center vs. restaurant manager) independent *t* test and then still found a significant main effect (see Figure 6), *t*(62) = 3.94, *p* < 0.001, and Cohen’s *d* = 0.98. People who strive to be a competent role (restaurant managers; *M* = 3.31, SD = 1.84) exhibited more consequentialist tendencies than those who aspire to be warm role (mental health center receptionists; *M* = 1.81, SD = 1.12).

In order to determine whether perceived external expectation mediated the effect of perceived target social roles on moral judgments, we conducted a 10,000-iteration simultaneous mediation bootstrap analysis using the PROCESS macro according to the procedures recommended by Preacher and Hayes (2008).

As expected, target social role positively perceived external expectation, *B* = 0.47, SE = 0.11, *p* < 0.001, and 95% CI [0.25, 0.70]. There was a significant indirect effect of target social roles on moral judgments through perceived external expectation, *B* = 0.27, SE = 0.07, *p* < 0.001, and 95% CI [0.14, 0.43] (see Table 1).

**TABLE 1:** Model estimation results for mediation of perceived external expectation effect on target role to moral judgment.

Predictor	Model 1: (DV: moral judgment)		Model 2: (DV: perceived external expectation)		Model 3: (DV: moral judgment)	
			
	*B*	*t* (64)	*B*	*t* (64)	*B*	*t* (64)
Target role	0.45	3.94***	0.47	4.23***	0.61	1.69
Perceived external expectation					0.54	5.19^∗∗∗^
*R*^2^	0.20		0.224		0.445	

The coefficients are standardized coefficients ***p < 0.001.

This provides further evidence that people indeed take into account the expectations of the outside world when making moral judgments. They engage in self-presentation of their moral judgments to ensure that the outside world receives information about the personality traits that they want to convey.

## General Discussion

### Moral Judgments as Signals of Personality Traits

Study 1 and Study 2 confirmed that people will infer personality traits based on others’ moral dilemma judgments and will also speculate about others’ moral judgments based on their personalities. Specifically, Study 1 showed that people see those who make deontological moral judgments as having personality characteristics on the warmth dimension and those who make utilitarian moral judgments as having personality characteristics on the competence dimension. Study 2 found that people think that a person who fits the warmth dimension will be inclined to make deontological moral judgments, whereas a person who fits the competence dimension will be inclined to make utilitarian moral judgments. According to these results, we can surmise that each type of moral decision has an underlying social signaling value.

In addition, Study 1 found that individuals with deontological moral judgments were rated positively as a whole. This result is also consistent with previous studies. In fact, moral intuitions often align with deontology. For example, people who made deontological decisions in moral dilemmas were rated as more empathetic and moral (Uhlmann et al., 2013). According to Kreps and Monin (2014), those who expressed utilitarian views were considered less moral than those who expressed deontological views, and they were sometimes even considered less moral than those who expressed no clear views. In a series of five studies, Everett et al. (2016) found that people who make deontological judgments are more likely to be selected as social partners, are considered more moral and trustworthy, and are more trusted in economic games. In a study by Sacco et al. (2017), participants reported liking and trusting deontological decision makers. Deontological decisions had an even greater effect on perceived trustworthiness than on liking, and trust was crucial for group cooperation. Researchers such as Everett et al. (2016) argue that these findings provide empirical support for the selection of moral intuitions and that deontological judgments confer an adaptive function by increasing the likelihood that a person will be chosen as a partner. Thus, deontological moral intuition may represent an evolutionarily prescribed *a priori* condition that is realized through partner selection mechanisms. This similar trend has been found in the context of China and other countries, suggesting to some extent that the sociocultural connotations of ethics may be consistent across cultures.

However, in some cases, utilitarianists are preferred to deontologists. According to previous studies and theories, it is well known that utilitarian judgments are often the result of slow, deliberative cognitive processes and they maximize revenue. In real life, when people choose social partners, what they value most about others may vary from relationship to relationship. When choosing an emotional partner, a deontologist may be viewed most favorably, but when choosing a doctor or lawyer, a utilitarian might be preferred.

### How Does Social Perception Affect Moral Judgment?

Moral behavior, for example moral judgments, can also be regarded as a form of self-presentation and impression management. Based on this idea, Study 3 and Study 4 examined people’s moral judgments in situations where they were aware of being perceived by others. We found that when people perceived that they needed to be in a social role that conformed to the characteristics of the warmth dimension, their moral judgment was inclined to deontology. When people needed to be in a social role that conformed to the characteristics of the competence dimension, their moral judgment was inclined to utilitarianism. Study 4 found that this association was mediated by perceived external expectations. These findings suggest that social cognitive factors have an impact on people’s moral judgment. Thus, a social cognitive perspective on moral judgment can perhaps address the limitations of the information and processing models of moral judgment. Social perception can be regarded as a kind of information, but it is different from the information emphasized in these other models, such as information related to moral events themselves and the agent’s intentions. Instead, information from social perception comes from the external environment.

How do we comprehend the results? When moral judgment occurs in a social context, it is no longer a private decision. However, few researchers have paid attention to social influences on moral judgment. From the perspective of evolutionary psychology, moral judgment and moral norms are derived from the strategic interaction among group members who experience interest fusion and conflict (Krebs, 2008). Considering that the human sense of morality is thought to have developed partly to promote social cooperation in groups, moral judgment that is sensitive to social perception is adaptive. The assumption is that when people believe that actions (judgments) contain rich information about others’ moral qualities, they use this information to obtain their own objectives. Perspective-taking or “mind-reading” abilities are invaluable tools in strategic interactions (Krebs, 2008). They enable people to construct cognitive representations of others and store those in mind; to look at events from the perspective of others and understand others’ thoughts, feelings, and plans; and to imagine how others will respond to their actions (Selman, 1980).

Krebs (2008) argued that the evolutionary mechanism of dynamic strategic interactions between communicative individuals explains the moral judgments that group members make, how they choose to accept or reject these judgments, and how certain moral judgments are copied and repeated enough to constitute cultural norms. Krebs proposed a simple model to describe the relationship between biological and cultural processes in the evolution of ethics. Every time an individual expresses a moral judgment of another individual, the receiver decides to accept or reject it depending on evolved information-processing and decision-making mechanisms. Senders (and receivers and observers) tend to repeat what others have accepted, and they avoid repeating what is not accepted. In this way, the process not only forms feedback but also contributes to the formation of a culture’s ethics. Therefore, moral judgments, which were found in this study to be sensitive to social perceptual factors, can also be seen as playing a role in promoting or maintaining moral norms.

### Limitations and Future Directions

This study has some limitations. Firstly, the sample size in Studies 2, 3, and 4 are relatively small and are not heterogeneous enough, and samples in Studies 2 and 4 are not very gender-balanced. Experiments on related topics in the future need to increase the sample size and try to use a more representative sample, with regard to age, sociocultural background, and gender. Second, social perception was studied only in terms of the dimensions of warmth and competence. However, moral judgments are influenced by many other pieces of information, which can be further investigated. For example, there are many personality traits that can be viewed as “warm” and “competent.” However, in Study 1, the scores of warmth dimension and competence dimension were obtained by averaging the score of four warmth traits and five competence traits, respectively. Participants in Study 2 were provided with five words of different dimensions when constructing a character with core characteristics, and it is unknown which exact words or traits the participants adopted to make the final prediction of the agent’s moral judgment. Therefore, future researchers can look for the connection between moral judgments and personality traits that are more elaborate and specific than the dimensions of social perception evaluated in the current study.

It needs to be emphasized that this study addressed the relationship between moral judgment and personality traits at the level of interpersonal perception. What we learned is that people make assumptions about the judgments that people will make based on certain personality traits, and their moral dilemma decisions are accordingly adjusted when they are in the context of specific requirements. The issue is not whether a person actually has these traits, or whether people with these traits actually make certain kinds of judgments; rather, it is social perception that matters. Therefore, whether they do so or not in actuality is unclear, and this is a topic that can be explored in future research.

## Data Availability Statement

The raw data supporting the conclusions of this article will be made available by the authors, without undue reservation.

## Ethics Statement

This study was approved by the Ethics Committee of the Central China Normal University. The patients/participants provided their written informed consent to participate in this study.

## Author Contributions

WJ and MP proposed the research, analyzed the experimental results, and wrote the manuscript. WJ designed and carried out the experiments. Both authors contributed to the article and approved the submitted version.

## Conflict of Interest

The authors declare that the research was conducted in the absence of any commercial or financial relationships that could be construed as a potential conflict of interest.
