# Supplementary material for: The Effects of Social Perception on Moral Judgment
Source: Front Psychol. 2021 Mar 23;11:557216. doi: 10.3389/fpsyg.2020.557216 (PMC8023274; doi:10.3389/fpsyg.2020.557216)
Supplement: Supplementary file 1 [file Table_1.DOCX]

**Supplementary Table on effects of Social Perception on Moral judgement**

**Authors**

Wen Ying Jin^1,2^, Ming Peng ^1,2,*^

**Affiliations**

1.School of Psychology, Central China Normal University

2.Key Laboratory of Adolescent Cyberpsychology and Behavior (Central China Normal University ), Ministry of Education

^*^**Corresponding author(s)**

Ming Peng

E-mail addresses: pengm2015@mail.ccnu.edu.cn

**Data**

The data is presented trough combined information as tables and figures. We measured the rating of the personalities of subjects who hold different moral judgments (Table 1), speculation on the moral judgment tendency of people in different social cognitive dimensions (Table 2), participants’ rating the importance of warmth and competence dimensions for both occupations in pretest (Table 3), the moral judgment scores of three moral dilemmas in two different social role groups (Table 4), participants’ rating the importance of warmth and competence for both social roles in the formal experiment in Study 3 (Table 5), moral judgment scores for moral dilemmas in different occupational role groups in Study 4 (Table 6). And linear regression (Table 7) and path analyses (Fig. 1) were carried out to understand the mediation effect of perception of others’ expectations.


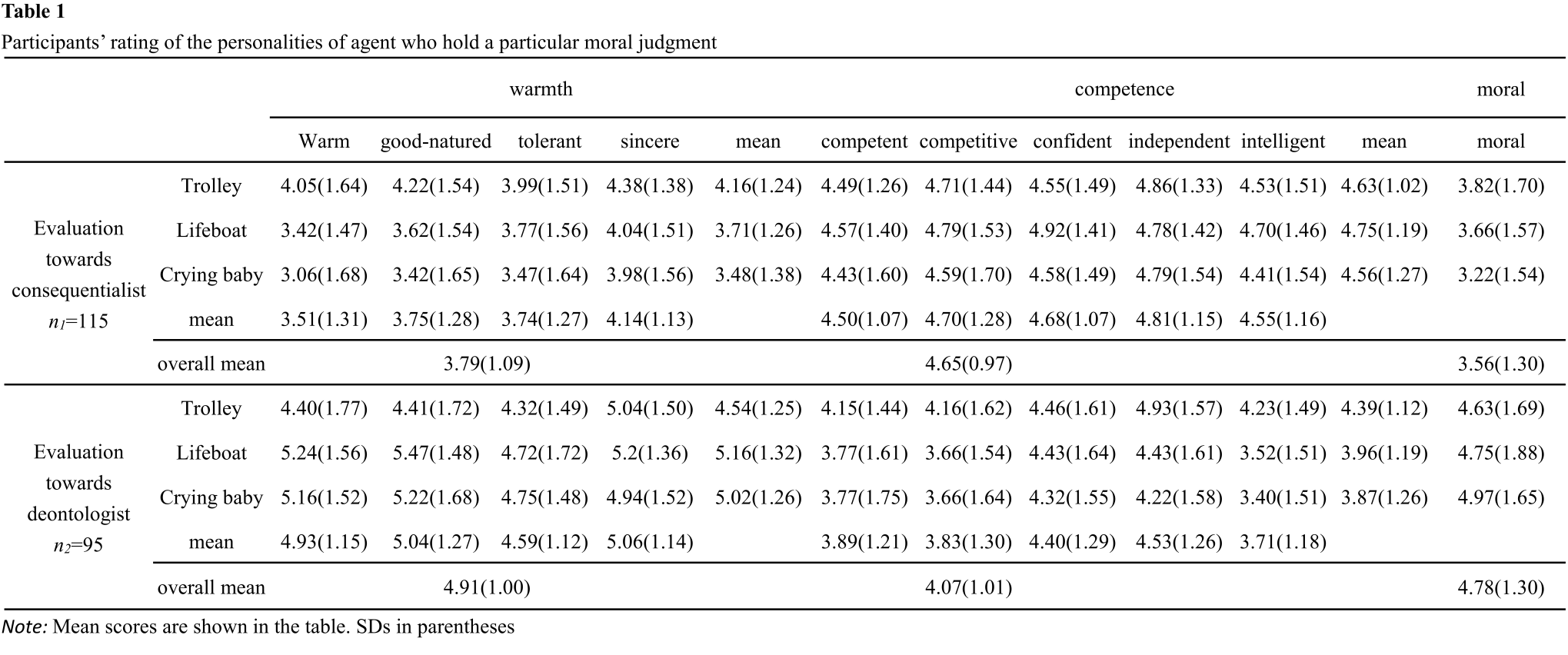


**Table 2**

Speculation on the moral judgment tendency of people in different social cognitive dimensions

|  | Speculations towards people in the warm dimension  *n_1_* =23 | Speculations towards people in the competen dimension  *n_2_* =22 |
| --- | --- | --- |
| Trolley | 3.91(1.73) | 4.91(1.90) |
| Footbridge | 3.35(1.61) | 5.64(1.26) |
| Lifeboat | 1.70(1.26) | 3.18(1.71) |
| mean | 2.99(0.95) | 4.58(1.12) |

*Note*: Mean scores are shown in the table. SDs in parentheses

In Study 3, participants firstly were asked to rate the importance of warmth and competence in two occupations in order to determine their suitability as priming social roles in the experiment. They rated the importance scores on scales from 1 (not at all important) to 7 (very important). The detailed scores in pre-test are listed in Table 3.

In the formal experiment, participants needed to work hard to get an offer in an interview simulation. Each job they applied for corresponded to a social character whose core quality was warmth or competence, and the interview process involves answering judgement about moral dilemmas. They in fact had to rate to what extent they thought that it was appropriate to carry out a utilitarian behavior in each scenario. Participants used a scale ranging from 1 (not at all appropriate) to 7 (very appropriate), with higher scores being closer to a utilitarian judgment and lower scores being closer to a deontological judgment. The detailed rating scores are listed in Table 4.

Finally, we re-examined the participates' ratings of the importance of warmth and competence in the two occupations. They rated the importance scores on scales from 1 (not at all important) to 7 (very important). The detailed scores are listed in Table 5.

**Table3**

Participants’ rating importance of warmth and competence for two occupations in pretest.

| Importance  *n* = 45 | Receptionist of Mental Health Center | Restaurant Manager |
| --- | --- | --- |
| Warmth | 6.53(0.73) | 5.20(1.18) |
| Competence | 5.09(1.35) | 6.51(0.59) |

*Note*: Mean scores are shown in the table. SDs in parentheses.

**Table4**

Judgement of moral dilemmas in different social role groups.

|  | Judgement on moral dilemmas |
| --- | --- |
| Receptionist of Mental Health Center  *n_1_*=34 | 2.96(1.35) |
| Restaurant Manager  *n_2_*=37 | 3.82(1.36) |

*Note*: Mean scores are shown in the table. SDs in parentheses.

**Table5**

Importance rating of warmth and competence for two occupations in formal experiment.

| Importance | Receptionist of Mental Health Center  *n_1_* = 34 | Restaurant Manager  *n_2_* = 37 |
| --- | --- | --- |
| Warmth | 6.41(0.89) | 5.20(1.18) |
| Competence | 5.53(1.21) | 6.51(0.59) |

*Note*: Mean scores are shown in the table. SDs in parentheses.

In Study 4, participants were asked to accomplish the task of job applying like in Study 3. We again measured participants’ moral judgments in the context of different social perception interventions and only one dilemma (Footbridge) was used here. Their detailed moral judgement scores are listed in Table 6.

**Table6**

Moral judgment scores for moral dilemmas in different social role groups.

|  | Footbridge |
| --- | --- |
| Receptionist of Mental Health Center  *n_1_*=32 | 1.81(1.12) |
| Restaurant Manager  *n_2_*=32 | 3.31(1.84) |

*Note*: Mean scores are shown in the table. SDs in parentheses.

| Predictor | Model 1: (DV: Moral Judgement ) | | Model 2:(DV: Perceived External Expectation ) | | Model 3: (DV：Moral Judgement) | |
| --- | --- | --- | --- | --- | --- | --- |
|  | *B* | *t*(64) | *B* | *t*(64) | *B* | *t*(64) |
| Target role | 0.45 | 3.94^***^ | 0.47 | 4.23^***^ | 0.61 | 1.69 |
| Perceived External Expectation |  |  |  |  | 0.54 | 5.19^***^ |
| *R^2^* | 0.20 | | 0.224 | | 0.445 | |

In addition, Study 4 added a cognitive question of participants on the recruiters' ideas at the end. We asked: “...... and what do you think the recruiter is looking for in your response to that dilemma question?”Then participants should rate it on scales from 1 (not at all appropriate) to 7 (very appropriate), with higher scores being closer to the deontological judgment and lower scores being closer to the consequentialist judgment.Multiple regression analysis was used as a test of mediation. The two roles (one requiring warmth, one requiring competence) were coded as dummy variables. Three regression models were established. We found that perceived external expectation fully mediated the effect of perceived target social roles on moral judgments (Table 7).

**Table 7**

Model Estimation Results for Mediation of Perceived external expectation Effect on Target Role to Moral judgement

*Note:* The coefficients are standardized coefficients. ^***^*p* < .001

**Acknowledgments**

This research was funded by the MOE(Ministry of Education in China) Project of Humanities and Social Sciences(No.18YJC90018)

**Competing Interests**

The authors declare that they have no known competing financial interests or personal relationships which have, or could be perceived to have, influenced the work reported in this article.

**References**

[1] Jin Wenying, Peng Ming. The effects of Social Perception on Moral judgment. .
